# Supplementary material for: Are carbon emissions trading and green financial instruments synergistic? -Comprehensive quantitative research based on content analysis
Source: PLoS One. 2024 Mar 7;19(3):e0298601. doi: 10.1371/journal.pone.0298601 (PMC10919601; doi:10.1371/journal.pone.0298601)
Supplement: S1 Data — (ZIP) [file pone.0298601.s001.zip › Data/地级市Z_F工作报告.zip!/╣π╢1⁄2/╓╨╔╜╩╨/└┤╫╘╣1⁄2╓┌║┼í╛┬φ┐╦╩2╛▌═°í┐_2013.docx]

2012年工作回顾

2012年是全市上下奋勇前进夺取重要成绩的一年。面对国内外严峻复杂的经济形势，面对非同寻常的各种考验，市政府在市委的坚强领导下，与全市人民一道，围绕率先加快转型升级、建设幸福和美家园的核心任务，千方百计稳增长，攻坚克难促转型，真抓实干惠民生，实现经济社会平稳较快发展。

经济质量效益得到新提升。全市生产总值2441亿元，增长11%，总量居全省第5，增速居珠三角第2，人均生产总值77527元。工业增加值1291.5亿元，增长14.5%。服务业增加值1025.2亿元，增长7.1%。固定资产投资893.4亿元，增长16.5%。社会消费品零售总额809.3亿元，增长10.3%。公共财政预算收入201.9亿元，增长10.2%。出口总值246.4亿美元，增长0.4%。实际利用外资8亿美元，增长10.1%。“三个一百”政策成效明显，引进一百投产项目产出强度增至844万元/亩，为2010年全市平均产出强度4.5倍，内资百强企业85%拥有自主品牌，百家外商投资企业及来料加工企业转型升级目标顺利实现。单位建设用地二三产业增加值、全员劳动生产率、平均立项投资额分别上升10%、11.4%、60.6%，万元生产总值能耗下降3.9%。实施《珠三角规划纲要》年度考评全省第4，比上年提升3个位次。

产业结构调整实现新突破。三次产业结构调整为2.5:55.5:42。先进制造业增加值占规模以上工业增加值比重33%，现代服务业占服务业比重61.5%，农业总产值增长2.2%。高新技术产品产值占规模以上工业比重提高1个百分点。成为省数控一代机械产品创新应用示范市，风电装备、新能源汽车、光电装备成为省市共建战略性新兴产业基地，健康科技产业成为国家创新型产业集群首批试点。市属国有经济结构布局优化。1万多家个体工商户转型为企业。4个省级产业转移园区累计开发4.5万亩。金融业增加值增长13.4%，境内外上市公司21家。实现省科技特等奖零的突破，发明专利授权增长31%。引进2家省级创新科研团队，新增4家国家重点实验室分支机构、1家院士工作站、7家博士后创新实践基地，培养引进各类人才4.7万人。

城市基础建设迈出新步伐。翠亨新区发展总体规划、建设方案、管理架构获省批准，进入实质建设阶段，翠亨国际旅游小镇项目开工。深中通道、深茂铁路等规划选线落实，古神公路二期、横二线东段、纵二线南段、纵四线、中山港大桥扩建等交通项目动工，105国道改造、广珠中线二期、十水线、东部快线等加快建设，福源路、广珠西线高速三期等建成通车。新建改建5个公交枢纽站，新投放192辆新能源公交车和出租车，实现中山通IC卡乘车市域全覆盖，15条跨市公交线路有效对接，中心城区公交出行分担率提高到23%。嘉明电厂三期等电力项目加快推进，中山积水潭骨科医院等卫生项目有序开展。启动秀美村庄建设，4个镇、15个村新增为省宜居示范镇村。改造危桥涵59座，建设农路硬底化195公里。获全省“三旧”改造奖。通过国家环保模范城复检。整治内河涌210公里，岐江河水环境生态保护区加快建设。中心城区雨污分流工程全面启动，新增污水处理能力11万吨/日，主要污染物总量减排考核居全省前列。新种与提升道路绿化400公里，新建与改造绿地面积71万平方米，城市人均公共绿地面积11.9平方米，森林覆盖率19.4%。建设绿道280公里，新投放3080辆公共自行车，使用频率居全国前列。

人民群众生活有了新改善。全市公共财政预算支出增加额9成以上用于民生保障。十件民生实事全面完成年度目标。城镇居民人均可支配收入3.1万元，农村居民人均纯收入1.9万元，分别增长12.4%和12.6%，高于生产总值和财政收入增速。新增就业5万人，城镇登记失业率2.3%，劳动力转移就业考核连续3年全省前三。扎实创建国家级创业型城市，建成11个创业孵化基地。社会保险城乡一体化水平提高，城镇职工社保总参保728.5万人次。城乡低保标准提高到每人每月430元，完成405户低保低收入家庭危房改造，发放临时价格补贴795万元。率先建成社会救助网上协同平台，为3万户老人新安装应急呼叫装置，成为全国养老服务标准化试点市。筹建保障性住房4333套，超额完成省任务。新建平价商店93家，居民消费价格涨幅2.3%。开展农村（社区）文化室高标准全覆盖工作，建成中山漫画馆。各类教育全面发展，高考录取率连续7年全省第一，校车安全实现全程信息化监管。推进公共卫生服务均等化项目，公办基层医疗机构全面实施国家基本药物制度和零差率销售，食品药品安全监督进社区。体育惠民广泛开展。蝉联全国全省双拥模范城。“双到”扶贫和对口援建成果巩固提高。

社会建设管理取得新成效。成立市镇两级社会工作委员会，建立社会发展监测预警机制。农村社区建设“2＋8＋N”模式等3项工作入选省社会创新观察项目。构建枢纽型社会组织，社工队伍及其本土化建设加快。全民修身行动成效显著，建成修身学堂650多间，高分通过全国文明城市测评。建设法治广东示范市，率先设置行政复议镇区受理点。广泛开展全民普法，人民调解成功率98.3%。城乡户籍统一登记为居民户口，人口计生工作机制不断完善。异地务工人员积分服务向住房保障延伸，1.4万人积分入户或入读公办学校。“三打”行动成果显著，“两建”工作扎实开展。实施全民治安工程，妥善处置突发事件，综治、信访、应急管理等工作保障了社会稳定。开展企业安全生产标准化建设，各类事故宗数下降，直接经济损失减少14%。台务、民族宗教、统计、审计、国防建设、妇女儿童、老龄、残疾人、气象、人防、档案、方志等各项工作取得新成绩。

行政服务效能得到新提高。出台支持中小微企业发展、鼓励工业投资、提升传统产业、发展新兴产业等一揽子政策，开展“五个一”暖企行动，采取150多项服务措施，为企业和基层解决一批实际困难。外事侨务工作获外交部“服务国内发展突出贡献奖”。坚持科学民主依法决策，办理人大代表建议137件、政协提案230件，满意及基本满意率100%。简政强镇事权改革稳步推进，下放事权1400多项。建设广东省网上办事大厅中山分厅，深化政务公开。完善电子监察系统，廉政勤政和反腐败工作加强。实行市属行政事业单位资产标准化管理，一般性行政经费支出压减5%，成为全省政府绩效管理试点市。

各位代表！回顾过去一年，我们深刻体会到：必须勇于克难奋进，积极应对稳增长促转型的繁重任务，紧紧咬住既定目标，众志成城、迎难而上，力促经济持续健康发展。必须善于转型升级，决不因为困难而放慢结构调整步伐，坚持以产业转型为引领、城市转型为核心、社会转型为支撑、政府转型为保障，力促加快转变发展方式。必须基于以人为本，强化人民利益在发展中的核心地位，越是困难时候越要保障民生，力促不断增进人民福祉。必须立于统筹发展，推进经济社会协同、区域发展均衡、人与自然和谐、新老中山人和睦，力促城乡全面协调发展。这是全市干部群众共同创造的宝贵经验，必须始终坚持、不断发展。

回顾过去一年，我们倍感成绩来之不易。这是省委、省政府和市委正确领导的结果，是市人大及其常委会和市政协监督支持的结果，是全市干部群众团结奋斗的结果，是海内外各界关心帮助的结果。在此，我代表市政府，向全市广大工人、农民、知识分子、企业家、干部群众，向各民主党派、工商联、各人民团体、无党派人士和社会各界人士，向驻我市解放军和武警官兵、人民警察，向所有关心支持中山发展的老领导、老同志、港澳台同胞、海外侨胞和国内外友人表示衷心感谢！

2013年工作安排

2013年是全面贯彻党的十八大战略部署的开局之年，是我市为全面建成小康社会夯实基础的重要一年，做好今年工作意义重大。当前，我市正处于大有作为的重要机遇期，也处于转型升级的爬坡越坎期。国内外经济形势复杂多变，我市发展仍存在许多困难和问题。经济增速放缓，保位争先压力加大；一些重点项目推进不快，发展后劲不足；基础设施薄弱，城市功能亟待提升；社会矛盾凸显，转型发展任重道远。我们既要充分看到积极因素，坚定发展决心；又要充分估计困难挑战，加强战略谋划。今后几年，要更加注重发展质量效益，更加注重统筹资源办要事，更加注重大企业大项目大平台拉动，更加注重增强发展内生动力，更加注重改革创新驱动，更加注重精细化管理。努力形成全市人民各尽其能、各得其所而又和谐相处的大好局面，努力创造更持续的发展、更和美的城市、更幸福的生活，努力在珠三角地区率先实现城乡居民人均收入比2010年翻一番。

2013年政府工作的总体要求是：以邓小平理论、“三个代表”重要思想、科学发展观为指导，全面贯彻党的十八大、习近平总书记视察广东重要讲话和省委十一届二次全会、市委十三届三次全会精神，牢固树立发展的统筹意识、人均意识、精品意识，加快结构调整，坚持改革创新，增进社会融合，努力改善民生，建设“三个适宜”幸福和美家园，为我省“三个定位、两个率先”总目标作出更大贡献。

2013年经济社会发展的主要预期目标是：生产总值增长10%，全社会固定资产投资增长15%，社会消费品零售总额增长12%，出口总值增长4%，公共财政预算收入增长10%，城镇居民人均可支配收入增长11%，农村居民人均纯收入增长12%，居民消费价格涨幅控制在3.5%左右，城镇登记失业率控制在3%以内，人口自然增长率控制在7.5‰以下，万元生产总值能耗下降3.9%，节能减排约束性指标完成省下达年度计划。贯彻省委、省政府保位争先工作部署，力争通过进一步转型升级，使增长性指标实现更好更快增长。

为实现上述目标，必须做好以下工作。

一、着力抓投资拓市场，促进经济持续健康发展

牢牢把握扩大内需这一战略基点，保持投资合理增长，注重投资的方向、结构和质量效益，挖掘释放消费潜力，拓宽国内国际市场，增强经济发展后劲。

加强重点项目建设。今年安排市重点项目98项，年度计划投资241.6亿元。健全重点项目动态管理、并联审批、要素保障、领导挂点、责任考核制度，形成开工一批、建设一批、竣工一批、储备一批的滚动开发格局，千方百计促进重点项目落地。加快重大产业平台、重大产业项目、重大核心自主技术产业化项目建设。

加大招商引资力度。瞄准战略性新兴产业、先进制造业、现代服务业进行重点招商。统筹招商资源，创新产业链对接型招商模式。修编产业发展导向目录，引进一批科技含量高、投资强度大、产业带动好的优质项目。探索成立外资投资基金，鼓励企业境内外并购重组。

积极扩大消费需求。支持企业开拓国内市场，创设内销品牌，参加国内会展。实施现代流通业发展十项工程，引进大型商贸流通企业，拓展中山产品全国销售网络，探索建设市外经贸合作平台，加快发展电子商务。编制全市商业发展规划，完善核心商圈和社区商业布点。引导房地产健康发展，建设城市综合体。增加城乡居民收入，增强居民消费能力。

提高经济开放水平。优化进出口结构，推动进出口增长。组织优势产品开拓国际市场。鼓励企业投保出口信用保险。建设重点进口商品分销平台，扩大资源性原材料进口。支持企业设立境外产销网络。促进加工贸易转型升级，争创综合保税区。

二、着力调整产业结构，提高经济发展质量效益

牢牢把握实体经济这一坚实基础，采取更加有利于实体经济发展的举措，构筑产品附加值高、质量效益好、要素资源占用少、能够支撑群众收入与经济同步增长的发展模式。

加强产业组织创新。完善先进制造业与现代服务业“双轮驱动”、工业化与信息化“两化融合”的新型产业体系。办好经济协作区，建设总部经济集聚区，吸引企业总部、区域总部、研发营销中心进驻。引导本地企业扎根中山“走出去”，培育生产在外、研发销售在内的企业组织。支持企业兼并重组、做大做强，鼓励大中小企业联盟协作。实施“新三百”计划，力争经过三年左右努力，培育10个百亿级企业、百个10亿级企业、10多个百亿级乃至若干个千亿级产业集群。

提升发展先进制造业。实现工业增加值增长10%。突破发展高端新型电子信息、半导体照明、新能源汽车产业，领先发展新能源、生物医药、高端装备制造产业，培育发展节能环保和新材料产业。推进风电装备制造、物联网和北斗导航产业、光电装备产业、软件产业等基地建设，发展游艇产业，打造省战略性新兴产业基地示范区。

大力振兴传统优势产业。实施传统产业改造计划，以设计、品牌、信息化、现代业态为手段，推动传统产业集群向现代产业集群提升。整合灯饰光源、家用电器、家具地板、纺织服装、金属制品、厨卫洁具等家居产业资源，打造“中山美居”区域品牌，建设“中山美居”体验与展销中心。推动资源依赖型、环境容量需求型企业异地转移发展。

加快发展现代服务业。实现服务业增加值增长10%。加快发展研发设计、检验检测、信息服务等科技服务业，继续推动企业研发设计等机构建设成为面向社会的独立法人。大力发展供应链物流、创新金融、结算中心等新型业态，积极发展健康卫生、智慧生活等幸福导向型产业。打造第二批5个市镇共建高端服务业集聚区，加快建设金鹰广场等服务业重点项目。推进翠亨国际旅游小镇等旅游业重点项目建设，发展城际旅游合作。

积极发展现代农业。提高农业综合效益，加强农业基础设施建设，推动农业集约化布局、专业化生产、社会化服务、产业化经营。推广农业科技，扶持花卉苗木、水果蔬菜、水产养殖等特色农业转型升级。发展农产品加工流通，培育绿色食品、无公害农产品，争创国家和省名牌产品。保障粮食安全。

三、着力推进创新驱动，增强经济发展内生动力

牢牢把握创新驱动这一核心战略，全方位推进科技创新、企业创新、产品创新、市场创新和品牌创新，发挥创新对转型升级的引领作用，建设创新型中山。

大力改善创新环境。健全促进创新的政策措施，完善政府为引导、企业为主体、社会资本参与的多元化科技投入体系，力争研发经费占生产总值比重达2.23%。强化产学研对接，引进高端研发机构。加强创新服务体系建设，鼓励公共技术平台开展核心和关键技术攻关。实施科技重大专项，推进生物大分子新药研发服务、大功率永磁同步电机、海上风机安装设备研制等项目。

发展科技型企业。实施科技强企计划，重点扶持成长性好的科技型企业。鼓励企业加大研发投入，组建各类工程技术中心，引进培育国家级创新平台。建设科技金融结合试点市，运用知识产权、股权质押等银行贷款方式，加大金融对企业创新支撑。重点推进无掩模光刻等核心自主技术产业化。

实施品牌标准专利战略。推进多层次品牌振兴工程，实现区域品牌与产业品牌、企业品牌齐头并进，支持省级以上名牌名标做大做强。推进技术标准工作，鼓励企业参与制定企业联盟标准、行业标准、国家标准。强化知识产权保护，推进知识产权快速维权中心建设，加强专利信息运用。

做好人才强市工作。发挥“1+20”人才政策效应，培养创新型人才，引进高层次人才，集聚紧缺适用人才，建设适应我市发展需要的人力资源大军。引进创新科研团队和科技领军人才，加强院士工作站、博士后工作站、创新实践基地、留学人员创业园建设。制定重点区域、行业、企业、项目人才扶持政策，努力使我市成为人才高地。

四、着力加强基础建设，提升城市发展综合实力

牢牢把握产城联动这一战略导向，构建产业转型升级与城市功能提升相互促进的协同机制，以城市功能完善增创发展优势，增强城市竞争力，打响中山品牌。

积极提升区位优势。抓住深中通道、深茂铁路等战略性项目建设的重大机遇，加强统筹规划，提升中山在珠三角乃至华南地区的战略地位。落实“211”和“603015”规划，加强与环珠江口航空港、深水港、轨道等重大站场交通衔接，推进中山北站等枢纽型交通设施规划。启动新中山港客运码头规划建设。

加强战略平台建设。整体规划、分步推进翠亨新区建设，制定政策体系，做好规划编制，创新开发模式，组建投资平台，开展起步区主干道等基础建设，抓紧围填海规划报批和实施工作，加快项目落地。规划建设岐江新城，打造高端总部经济区、生产服务区和金融商贸区。

加快完善交通体系。继续落实交通先行再先行、公交优先再优先战略，促进外拓内畅。推进深中通道广珠连接线、中江高速复线前期工作。实施“改造一环、建设二环、规划三环”的环市快速路建设计划，加快改造建设二环路、北外环路、105国道，推进建设沙港公路、纵四线、横四线西段和中山港大桥扩建。实施公交发展战略规划，加强交通组织管理，科学精细管控道路，大型项目动工先通过交通评估。

优化城市功能布局。出台主体功能区规划，统筹公共设施布局。改造提升主城区，加快东部、西北部、南部三个片区副中心规划建设。完善电网结构，加快能源项目和输变电工程建设。做好主城区和门户地区城市设计，打造岭南特色示范工程。加强历史文化名城保护，发掘保护一批文化街区和古村落。完善数字城管信息系统，推进云计算技术广泛应用。

五、着力强化统筹兼顾，打造市场经济良好环境

牢牢把握优化秩序这一重要责任，正确处理好政府与市场的关系，更加尊重市场规律，更好发挥政府作用，营造法治化国际化营商环境。

加强资源统筹利用。市财政安排产业扶持资金5亿元。坚持全市一盘棋，统筹招商力量、项目安排、用地指标、环境容量、电力供应、扶持资金等，向重点企业和优质项目聚集。建立项目流转内部超市，鼓励项目从产业规划、资源整合、利益共享出发在镇区间流转。推进土地资源、金融资金、产业资本融合发展。

加大帮扶企业力度。当好企业初创期的“保姆”、成长期的“保安”、成熟期的“保镖”。加强扶持政策协调配套，落实中小微企业优惠政策。实行领导干部联系企业长效机制，一企一策重点帮扶。健全企业综合服务体系，推广小榄生产力促进中心经验，发挥工业设计、管理咨询、融资服务、人才培训、企业孵化等服务平台作用。保护和弘扬企业家精神。

规范市场经济秩序。巩固“三打”成果，推进社会信用体系和市场监管体系建设。构建公共联合征信系统，实行企业信用分类监管，做好小榄、古镇综合试点和信贷行业试点。健全重点领域和重点行业市场监管制度，做好石岐综合试点和药品行业试点。推进质量强市建设。加强经济运行监测，提高经济防抗风险能力。

强化金融服务支撑。推进城乡金融服务一体化综合改革。发展地方金融，建设专业镇金融服务中心和村居金融服务站，扩大小额贷款公司、村镇银行等金融机构覆盖面。促进企业境内外上市，发展创业投资行业，推进“新三板”试点。丰富金融业态，推动基金、债券、信托、期货发展。加强与海外金融资本合作，对接穗深等区域资本市场。规范民间融资行为，防控金融风险。

提高土地利用效益。强化统筹规划、征收、开发、出让、使用的土地管理机制，依法清理闲置低效和供而未用土地，提升土地产出率。加快“三旧”改造，盘活存量用地。加强土地储备管理，创新土地开发模式。开展城乡土地综合整治试点。

六、着力发展生态文明，建设更加宜居美好家园

牢牢把握生态文明这一绿色理念，把创建国家生态文明示范市放在突出位置，倡导低碳增长模式和消费方式，建设天蓝、地绿、水净的美丽中山。

优化生态格局。促进生产空间集约高效、生活空间宜居适度、生态空间山清水秀。开展生态区域控制规划，合理控制城市开发强度，优化调整用地布局，加强山体保护与滨水岸线塑造，建设山、河、海、城、乡有机统一的现代生态山水名城。

绿化生活空间。打造适应现代城市发展需要的绿色空间，实现“三年绿化大提升”目标。加快主城区和镇区中心绿化升级，建设生态景观林，广泛植树造林，形成多层次绿化带。推进森林进城围城工作，建设一批森林公园。完善慢行系统规划设计，建设镇区公共自行车系统。

保护水体环境。建设国家水利现代化试点市，促进人水和谐。推进岐江河水环境综合整治、雨污分流等重点工程。完善生活污水处理厂及配套管网建设，火炬区、小榄、古镇、横栏、南头等扩建项目投入运行，坦洲、阜沙、东凤、南朗等项目加快建设。实施最严格水资源管理制度，加强用水总量控制，严格取水许可和入河排污口监管。加快推进抗咸工程，形成水安全长效机制。

建设秀美村庄。构建新型城乡关系，推进城乡规划、基础设施、公共服务一体化。按照因地制宜、环境优美、设施完善、特色鲜明的要求，编制秀美村庄建设规划，重点实施村容整洁工程。建好市镇两级示范点和名镇名村，创建第四批宜居示范镇村。

强化节能减排。推进工业生态化，坚决淘汰落后产能。推行清洁生产，推广低能耗低排放生产技术，创建资源节约型、环境友好型示范企业。抓好建筑、交通、商贸、公共机构和农村等非工领域节能。严格执行污染物总量减排，促进农业源、城镇污水、工业锅炉和机动车主要污染物专项减排。强化水、土壤、大气等重点领域污染防治，加强重金属和固体废物污染综合防治。

七、着力突破重点改革，完善科学发展体制机制

牢牢把握深化改革这一动力源泉，把改革作为发展的最大红利，推进重点领域和关键环节改革，构建有利于要素高效配置、运行科学有效、激活发展潜力的制度安排。

深化经济体制改革。开展要素配置管理改革，以资源投放绩效评估为核心，确保向优质项目倾斜。开展财政综合管理改革，完善市镇两级分配制度，探索产业扶持资金股权投资办法，增强财政资金放大作用。开展市属国有企业改革，促进股权多元化，引入优质战略投资者，实现多种经济成分高效聚合。开展投融资体制改革，建立投资主体多元化、融资渠道多样化的新型体制，引导民间资本参与政府投资重点项目建设，充分发挥社会资本作用。开展商事登记制度改革，实现市场准入宽进严管。

加强农村综合改革。推进“政经分离、政社分离”，逐步实现基层自治、经济管理、社会服务适度分离。完善农村股份合作制和村级统一核算改革，引导条件成熟的村（居）集体经济组织实行公司化改造。强化集体土地管理，促进农村土地集约节约利用。加强农村基层组织建设，维护农民合法权益。

开展医药卫生体制改革。以市民受益为目标，运用信息化和现代医院管理模式，建立居民健康档案信息化系统，再造卫生管理流程。关注薄弱环节，选准卫生资源增量投向，强化分级发展，促进优质卫生资源下移。深化公立医院改革，优化民营医院发展格局。探索医疗保障支付制度改革，引导基层首诊和分级诊疗。

推进行政体制改革。完善简政强镇事权改革配套措施，加强下放事权业务指导，健全镇区综合执法机制。推进事业单位分类改革。促进审批制度标准化规范化，健全网上办事大厅数字认证、网上缴费、移动办公等功能。探索设立行政审批事项公示听证制。

积极开展区域合作。贯彻《珠三角规划纲要保障条例》，狠抓重要指标、重点任务、重大项目，确保“四年大发展”总验收取得好成绩、“九年大跨越”取得新突破。推进珠中江深度合作，提高基础设施互通和民生项目互联水平。加强与港澳穗深合作。开展我市“十二五”规划中期评估。

八、着力改善民生保障，努力增进城乡人民福祉

牢牢把握服务人民这一根本宗旨，坚持多谋民生之利、多解民心之忧，做到为民办实事、惠民重实效，让人民群众共建共享改革发展成果。

完善社会保障体系。实施充分就业和创业富民工程，提高中等收入者比重，逐步解决市内相对贫困问题。做好高校毕业生、困难人员、特殊群体等就业工作，年内城镇新增就业5万人。健全社会保险制度，提高企业职工参保率，加强社保基金风险防控。做好住房保障工作。发挥市统一救助平台作用，完善社会救助体系，倡导社会互助。加强红十字会与慈善总会建设，发展妇女儿童与残疾人事业。

推进文化强市建设。深化全民修身行动“巩固年”工作，做好全国文明城市测评。加强公共文化设施建设，提升镇区文化活动中心水平，扶持基层业余文艺团队发展，完成国家公共文化服务体系示范项目建设。创新公共文化服务提供方式，鼓励社会资本进入文化领域。繁荣文化事业，发展文化产业，做大做强游戏游艺、文化创意和特色文化产业。

办好人民满意教育。全面实施素质教育，培养学生社会责任、创新精神和实践能力。促进基础教育优质均衡发展，推进统筹高中阶段教育。优化整合职业教育资源，打造具中山特色的高层次职教体系。发展高等教育，做好与澳门科技大学合作办学工作。完善全民终身教育体系。深化教育领域改革，提高教师队伍素质。优化教育结构，促进民办教育规范化、优质化、差异化发展。

提高人民健康水平。实施医疗质量、医德医风、医学人才三大提升工程。建设区域卫生信息平台，鼓励社会办医。提高社区卫生队伍素质，优化社区卫生服务。加强公共卫生与基本医疗服务体系建设，提高卫生应急防控处置能力。办好全民健身示范城市试点，完善体育公共服务体系，推动竞技体育科学发展。

全面发展社会事业。科学调控人口规模，促进人口长期均衡发展。做好免费孕前优生健康检查和异地务工人员计生服务工作，完善“惠一生”利益导向政策。用好侨乡优势，发挥外事侨务、港澳事务、台务服务发展的作用。做好民族宗教、国防建设、双拥、老龄、人防、气象、地震、档案、方志等工作。

九、着力创新社会管理，推进社会安定和谐善治

牢牢把握创新管理这一有效手段，营造安定善治、公平正义、均衡协调、普惠包容、充满希望的社会氛围，让新老中山人共享做中山人的尊严和荣耀，建设和谐中山。

完善社会管理体系。健全党委领导、政府负责、社会协同、公众参与、法制保障的社会管理体制。设立社会管理与创新专项资金，探索村（居）公共产品多元供给机制。形成社区工作人员、社工、志愿者、群众共同参与基层服务新格局，扩大社工覆盖面，加强社区服务中心建设，构筑管理有序、服务完善、文明祥和的社区生活共同体。创新市民“点菜”、公众评价的社会管理。

积极增进社会融合。坚持“新老中山人都是一家人”理念，率先破解城市二元结构问题，促进“三个共享”，维护发展新老中山人同心协力的良好局面。支持异地务工人员参与社会管理，评选“同是中山建设者”百佳异地务工人员。开展服务新中山人的民间互助和慈善公益活动。

培育发展社会组织。完善政策扶持、分类登记、等级评估机制。建设社会组织孵化基地，重点扶持工商经济、社会服务、公益慈善、群众生活类社会组织发展，强化工青妇等群团组织枢纽功能。推进政府向社会组织转移职能和购买服务，扩大社会力量对社会管理服务参与度。加强社会组织规范管理和行业自律。

深化平安中山建设。完善立体化治安防控体系，创建全国最平安城市。畅通群众诉求表达、利益协调、权益保障渠道，做好综治信访维稳工作。完善人民调解、行政调解、治安调解、司法调解相衔接的矛盾化解机制，构建和谐劳动关系。健全重大决策社会稳定风险评估机制，完善突发事件防控处置，提升应急管理水平。强化生产安全、社会安全监管，加强网络社会管理。

十、着力提升行政效能，建设为民务实清廉政府

牢牢把握人民满意这一本质要求，充分尊重民意，改进工作作风，提升政府创造良好发展环境、提供优质公共服务、维护社会公平正义的履职效能。

为民行政。坚持全心全意为人民服务，强化主动服务，提高服务质量，提升市民认可度。密切联系群众，凡是涉及群众利益的决策都要认真听取群众意见，凡是损害群众利益的做法都要坚决防止和纠正。及时了解群众所忧所急，把群众工作做实做透，对人民负责、受人民监督、让人民满意。

依法行政。坚持依法管理事务和行使职权，加强行政处罚自由裁量权标准化管理，做好行政复议。推进法治广东建设示范市工作，深入开展“六五”普法，加强法律援助，保障市民合法权益。自觉接受人大法律监督和政协民主监督，定期向市人大报告工作和向市政协通报情况，认真办理人大代表建议和政协提案，充分听取各民主党派、工商联、无党派人士和各人民团体意见。

高效行政。坚持务实高效和狠抓落实，提高政府执行力和公信力。加强政府绩效管理，开展履职效率、效能建设、服务效果、创新创优考评，推进工作提速增效。严格执行中央改进作风八项规定和省市要求，精简议事协调机构，减少会议活动文件。

廉洁行政。坚持精打细算和勤俭节约，从紧控制消费性消耗性支出，各部门公用经费预算零增长，降低行政成本。简化接待，严控出访。加强政务公开，推进网络问政。严格监察审计，实行对政府全口径预算审查监督。强化土地使用权出让、建设工程招投标、政府采购、产权交易、资金使用等关键环节反腐倡廉，实施廉洁中山建设五项工程。

经过广泛吸纳民意，今年我市继续集中力量办好十件民生实事。一是实施全民治安工程。创建平安村居、厂企、小区、校园、医院、出租屋、市场，建设网安警务室和网上报警岗亭，新建整合一类治安视频监控点1200个、高清治安卡口48个。二是做好综合治水工作。雨污分流中心城区主干管网开工建设32项、完成40公里，市政污水管网开工建设13片区、完成117公里，支管到户开工建设30个片区、完成10个片区。19个镇区完成第一批32个村（社区）雨污分流任务。推进岐江河水环境整治二期工程。三是提高道路畅通能力。实行市领导包干督导，加大力度打通镇际未连接的重点道路，确保年内打通一批、推进建设一批、规划落实一批。完善镇区至城区公交大站快线网络，试点开展公交小巴“招手即停”。四是加强食品药品管理。检测蔬菜农药残留不少于50万份，抽检食品及原料16类、药物品种1000批。做好“放心菜”生产基地、无公害生产基地、绿色食品生产基地等监督管理，推进标准化生产。五是提升医疗卫生服务。提高社会医疗保险待遇。严格控制医疗费用增长，减轻群众治病负担。镇级以上医院检验检查结果互认。加大对低收入群体重特大疾病医疗救助力度。六是积极发展学前教育。构建公办民办并举的学前教育公共服务体系，落实“惠民扩优”工程，提高幼儿教育质量，全市等级幼儿园比例达35%。七是推进助困养老服务。落实低收入群众临时价格补贴与物价上涨联动机制，最低生活保障标准年内增幅达11%。完成低保低收入和优抚对象500户危房改造。新建居家养老服务中心5家，对三无、五保、低保等独居老人免费提供上门家政服务，加快新社会福利院建设使用。八是促进农村和谐发展。积极解决问题突出村和相对贫困村问题。实施“一村一策、一户一法”，扶持相对落后村及低收入农户。加强农村集体资金、资产、资源管理，完成镇区资产监管和交易平台建设。九是优化积分服务管理。拓展异地务工人员享受基本公共服务的广度深度，积分制向住房保障和医疗保险延伸，简化申请审核流程。十是完善群众文体设施。加大公共文化体育设施免费开放或优惠开放力度，机关、企事业单位体育场地逐步向社会开放。扩大全民健身设施覆盖面，定期维修更换健身设施。
